# Effects of Phthalate Esters on *Ipomoea aquatica* Forsk. Seedlings and the Soil Microbial Community Structure under Different Soil Conditions

**DOI:** 10.3390/ijerph16183489

**Published:** 2019-09-19

**Authors:** Tingting Ma, Linwei Liu, Wei Zhou, Like Chen, Peter Christie

**Affiliations:** 1 Key Laboratory of Original Agro-Environmental Pollution Prevention and Control, Ministry of Agriculture/Tianjin Key Laboratory of Agro-environment and Safe-product, Tianjin 300191, China; ttma@issas.ac.cn; 2 Institute of Hanjiang, Hubei University of Arts and Science, Xiangyang 441053, China; 3 Key Laboratory of Soil Environment and Pollution Remediation, Institute of Soil Science, Chinese Academy of Sciences, Nanjing 210008, China; drpeterchristie@aol.com; 4 School of Civil Engineering and Architecture, Hubei University of Arts and Science, Xiangyang 441053, China; 2017121225@hbuas.edu.cn; 5 Shanghai Research Institute of Chemical Industry, Shanghai 200062, China; clk@ghs.cn

**Keywords:** water spinach, PAEs, 454 high-throughput sequencing analysis, phytotoxicity

## Abstract

Phthalate acid esters (PAEs) are the most frequently utilized synthetic chemical compounds worldwide. They are typical emergent contaminants and are currently attracting considerable concern due to their risks to plants, animals, and public health. Determining the vital environmental factors that affect the toxicity of target pollutants in soil is important for vegetable production and the maintenance and control of soil productivity. We investigated the influence of di-*n*-butyl phthalate (DBP) and bis(2-ethylhexyl) phthalate (DEHP) under different soil conditions on physiological changes in water spinach (*Ipomoea aquatic* Forsk.) seedlings and the rhizosphere soil microbial community. Supported by our former experiments in which we determined the representative concentrations that caused the most pronounced toxic effects, three experimental concentrations were studied including control soils without PAEs and spiked soils with either 20 mg DBP or DEHP kg^−1^ soil. The soil at all the three PAE concentrations was then adjusted to test two soil pH values, three levels of soil organic matter (SOM) content, and three levels of soil moisture content; thus, we completed 12 treatments or conditions simulating different soil environment conditions in greenhouses. After 30 days of cultivation, we analyzed the toxicity effects of two target PAEs on plant growth and physiological factors, and on soil microbial community characteristics. The toxicity of soil DBP and DEHP to the physiology of water spinach was found to be most affected by the soil pH value, then by SOM content, and least of all by soil moisture. The results of the 454 high-throughput sequencing analysis of the soil microbial community indicated that the toxicity of target PAEs to soil microorganisms was most affected by SOM content and then by soil moisture, and no clear relationship was found with soil pH. Under different soil conditions, declines in leaf biomass, chlorophyll a content, and carotenoid content—as well as increases in free amino acid (FAA) content, superoxide anion free radical activity, and hydroxyl radical activity—occurred in response to DBP or DEHP. Heavy use of chemical fertilizer, organic fertilizer, and high humidity led to the special environmental conditions of greenhouse soil, constituting the main conditions considered in this study. The results indicate that under the special highly intensive production systems of greenhouses, soil conditions may directly influence the effects of pollutant phytotoxicity and may thus endanger the yield, nutrient content, and food safety of vegetables. The combined studies of the impacts on plants and rhizosphere microorganisms give a more detailed picture of the toxic effects of the pollutants under different soil conditions.

## 1. Introduction

Since the previous century, endocrine-disrupting chemicals (EDCs) have aroused great concern globally due to their adverse effects on reproduction and development in both wildlife and humans [1,2]. The plasticizers phthalate acid esters (PAEs) are an important group of EDCs [3], and are colorless, oily liquids that are often added to impart flexibility and plasticity in a very wide range of consumer goods [3,4,5]. About six million tonnes of PAEs were produced worldwide in 2012, and their annual production and consumption in China have recently reached 45 million and 22 million tonnes, respectively [6,7]. Moreover, PAEs are not covalently bound to polymer molecules, and this can result in their widespread migration at significant quantities into the atmosphere, water, soil, sediments, and foods [8,9].

Di-*n*-butyl phthalate (DBP) and bis(2-ethylhexyl) phthalate (DEHP) are the two most common PAE compounds, and long-term exposure to low levels leads to teratogenic, mutagenic, and carcinogenic effects [10,11,12,13]. They are both included as potential EDCs in the priority pollutant list established by the US Environmental Protection Agency (USEPA) [14], and might enter crops and possibly the human body via the food chain [15,16]. In greenhouse vegetables and soils covered by plastic films—including both mulching film and shed plastic film—the highest sum of DBP and DEHP concentrations has been determined to be over 32 mg kg^−1^ in provinces such as Jiangsu, Guangdong, Shandong, and other urban areas of China [14,17,18,19,20,21]. The problem binding with microplastics and other derivatives has also added complications ever since the recognition of their risk [20]. The development of facility greenhouse production all over China is still occurring at high speeds, and the toxicity effects of PAEs in varied soil types requires more attention.

Xenobiotic phytotoxicity can be demonstrated by the production of morphological, physiological, and molecular stress responses by plants [22]. PAEs can induce germination inhibition, photosynthesis blocking, biomass and nutrition (vitamin C and soluble sugar) content decline, abnormal cell division, root morphology, and metabolism in vegetables [23,24,25,26,27,28,29]. Increases in the micronucleus rate, membrane permeability, oxidative damage, and genotoxicity in plants have also been detected [22]. The higher determined levels of PAEs and their concerning toxicity damage to plants has led to a great focus on PAE contamination control and other health-risk problems. Increasing evidence has confirmed the importance of plant-associated bacteria for plant growth and productivity [13]. On account of the high residual levels of either DBP and DEHP in soils, there is also growing concern over soil microbial community functional diversity reduction, microbial biomass carbon inhibition, soil basal respiration suppression, and soil enzyme activity loss [30,31,32,33]. Commonly determined soil properties include color, particle size, and mineralogical and chemical properties. Techniques such as mid-infrared (MIR) spectroscopy can be useful for the initial screening of samples [34,35,36], but more intricate analysis is often required to discriminate between samples. High-throughput sequencing has been used with impressive effect in the investigation of soil microbial and animal community structures for more than a decade [37,38,39]. It has become an increasingly important tool for the detection of microbial communities associated with the guts of animals from different soil types [40,41,42]. However, the toxicity of pollutants may be related to their concentration, form and distribution, mineral levels, soil organic matter (SOM) status, mechanical composition, and soil pH level. Therefore, there is a need to understand how DBP and DEHP pollution impact vegetable production and soil microbial communities in greenhouses, especially under different soil conditions.

The present study sought to address current knowledge gaps by investigating the influence of the vital soil factors of SOM content, pH value, and soil moisture on the phytotoxic effects of single DBP and DEHP on water spinach and soil microbial communities. Water spinach is a common vegetable with a short harvest interval that is intensively cultivated all over China. The foliar biomass, chlorophyll a content, carotenoid content, free amino acid (FAA) content, superoxide anion free radical activity, and hydroxyl radical activity were determined together with their impacts on soil microbial communities using high-throughput sequencing after incubation for 30 days. In our former study on lettuce, soil water and SOM contents were measured and compared, and the most effective pollutant concentration was screened, while no detailed investigation on soil microbial communities has so far been conducted. The present study was designed based on the more authoritative and refined analysis of high-throughput sequencing, as well as to compare the toxicity differences of the two target PAEs to other vegetables with lettuce. The treatments at different levels of pH, SOM content, and water content were set according to the actual soil conditions to simulate the different soil types in China. The results aim to provide recommendations on vital factors of different soil conditions affecting PAE ecotoxicity as well as suggestions on plastic film utilization in greenhouses under different soil conditions.

## 2. Materials and Methods

### 2.1. Standards, Assay Kits, and Reagents

DBP (99.1%) and DEHP (99.6%) certified reference materials (CRMs), assay kits (catalog number A052, inhibition and production of superoxide radical; A018, hydroxyl radical) and other analytical-grade reagents were purchased, and details of their use are as described in Ma et al. [29].

### 2.2. Test Soil and Plants

Detailed information about the selected soil can be found in Ma et al. [29]. The soil background concentration levels of DBP and DEHP were 0.037 ± 0.002 and 0.087 ± 0.004 mg kg^−1^ (dry weight (DW), freeze dry basis), respectively. Seeds of water spinach (*Ipomoea aquatica* Forsk.) were obtained from the Chinese Academy of Agricultural Sciences in Nanjing. The storage and sterilization of the seeds and the preparation methods of glassware were as described in Ma et al. [29].

### 2.3. Toxicity Tests

Clay pots, each containing 2.0 kg of soil, were prepared for investigation. Two soil pH levels (7.0 and 8.5), two levels of SOM content (2.0% and 4.0%), and two levels of soil water content (60% and 80% maximum water-holding capacity (WHC)), four groups at 0 PAE control, 20 mg DBP kg^−1^, and 20 mg DEHP kg^−1^ were designed, resulting in a total of 12 different treatments. The four test groups were set to simulate different soil types from different parts of China because variation in pH, soil organic matter content, and water content are the major differences found among actual soils. These treatments included (1) pH 7.0, 2.0% SOM, 80% maximum WHC; (2) pH 8.5, 2.0% SOM, 80% maximum WHC; (3) pH 7.0, 4.0% SOM, 60% maximum WHC; and (4) pH 8.5, 4.0% SOM, 60% maximum WHC. A pH of 8.5 is often observed in the soil of North China, the alluvial area of the Yangtze river basin in Central China, and the limestone soil of Southwest China. A maximum WHC of 80% in facility greenhouses is commonly seen, which is considered sufficiently wet (rather than leaking). There were four replicate pots for each treatment. The adjustments of soil pH values, target pollutants, and SOM (using carbonized peat moss) were performed as described in [29]. Following this, each soil sample (S1–S12) was mixed thoroughly, and the moisture contents were prepared by adding water to the appropriate weights before use, as shown in Table 1. The different water contents were maintained by weighing every day and water was added to 60% or 80% of the maximum WHC by gently blending after watering and trying to reduce moisture variation at different depths in clay pots. A total of 48 pots were used for plant cultivation for 30 days from 20 April to 19 May 2017, at 25 ± 0.2 °C under a 12-h day cycle with 4500 lux illumination. In each clay pot, five seeds were arranged in spiked soils. The harvested seedlings and soils were collected at the end of the cultivation period.

### 2.4. Quantitative Analysis of PAE Compounds

The analysis of soil DBP and DEHP concentrations in harvested samples, parallel samples, and whole procedure blanks, and the analysis of CRMs all followed the procedures described in Ma et al. [29] to ensure quality control. An isotopically labeled PAE, di-n-butyl phthalate-d4 (DBP-D4, 100 µg mL^–1^), was used as a surrogate in whole procedure blanks (recovery rates 88.3~92.7%). Recovery results of the two target PAE compounds in CRMs are listed in Table S1.

### 2.5. Determination of Physiological and Biochemical Indices

Shoots of all harvested water spinach in each pot were rinsed with tap water and then with deionized water before blotting dry with paper. The biomass (fresh weight (FW)) in each pot was also determined by weighing, and this was recorded immediately. The amounts of plant pigments were calculated using the equations of Ma et al. [16]. The FAA content was estimated following the method of Ma et al. [29]. Analyses of superoxide anion free radical activity and hydroxyl radical activity in plants were conducted using the corresponding assay kits after the formation of fresh plant supernatant following the instructions of Ma et al. [29].

### 2.6. DNA Extraction, Pyrosequencing, and Pyrosequencing Analysis

After microbial DNA extraction, the V4–V5 region of the bacterial 16S rRNA gene was amplified following the method of Hou et al. [43], and 454 pyrosequencing was conducted by Majorbio Biopharm Technology Co., Ltd., Shanghai, China. The 16S rRNA sequencing data, coverage index, and α-diversity indices Ace, Chao, Shannon, and Simpson were calculated with MOTHUR (1.37.4), with chimeric sequences identified and removed using UCHIME following the methods and guidelines of Hou et al. [43].

### 2.7. Statistical Analysis

All statistical analyses were performed using SPSS 18.0 (SPSS, Chicago, IL, USA). Principal coordinate analysis (PCoA) and heatmaps were plotted in R (3.3.2). Each value is the mean of four replicates ± the standard error of the mean (SEM). The symbol * means significantly different at the *p* < 0.05 level and ** means significantly different at the *p* < 0.01 level according to a Duncan’s multiple range test within each group as compared with the corresponding controls. A Mantel test was performed to assess the correlations between the Bray–Curtis dissimilarity matrix of the detected Operational Taxonomic Units (OTUs) and environmental parameters (target PAE compounds and different soil physiological characteristics) by using R package “vegan” (Table S2).

## 3. Results

### 3.1. Effects on Plant Biomass

**Figure 1:**
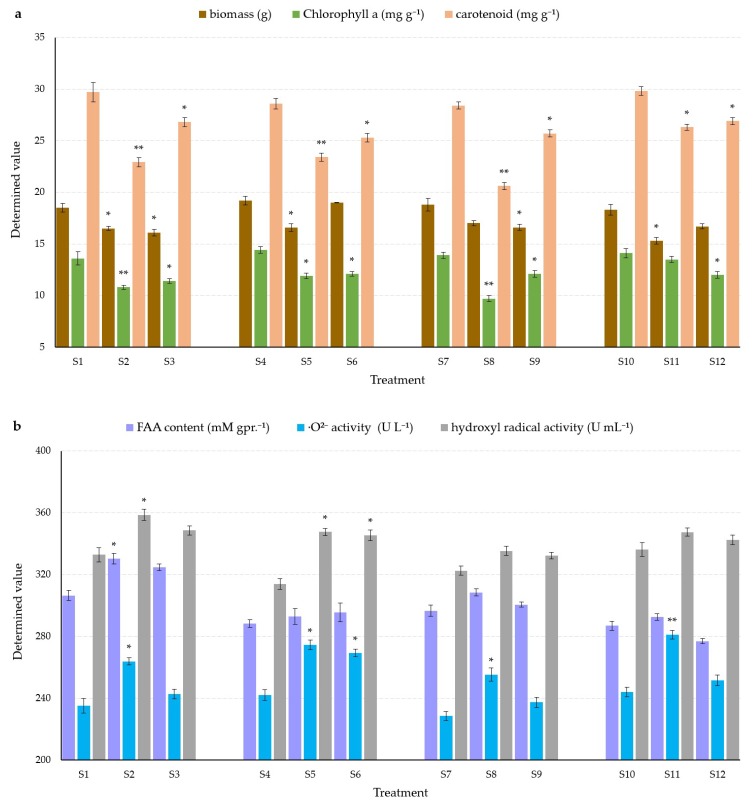
Effects of the target PAEs on physiological changes in (**a**) biomass (g), chlorophyll a content (mg g^−1^), and carotenoid content (mg g^−1^); and (**b**) free amino acid (FAA) content (mM per gram protein^−1^ (mM gpr.^−1^)), superoxide anion free radical activity (U L^−1^), and hydroxyl radical activity (U mL^−1^) of water spinach under different soil conditions. S1, S2, and S3 correspond to 0 control, DBP 20, and DEHP 20 mg kg^−1^, respectively, all at pH 7.0, 2.0% SOM, 80% maximum WHC; S4, S5, and S6 correspond to 0 control, DBP 20, and DEHP 20 mg kg^−1^, respectively, all at pH 8.5, 2.0% SOM, 80% maximum WHC; S7, S8, and S9 correspond to 0 control, DBP 20, and DEHP 20 mg kg^−1^, respectively, all at pH 7.0, 4.0% SOM, 60% maximum WHC; and S10, S11, and S12 correspond to 0 control, DBP 20, and DEHP 20 mg kg^−1^, respectively, all at pH 8.5, 4.0% SOM, 60% maximum WHC. Each value is the mean of four replicates ± SEM. * significantly different at *p* < 0.05; ** significantly different at *p* < 0.01 according to a Duncan’s multiple range test within each group compared with the corresponding controls

The toxicity of two target PAEs on biomass in water spinach under different soil conditions throughout the seedling growth period of 30 days is shown in Figure 1a. Plant biomass was inhibited in all treatments by the two target PAEs, and the decline appeared to be maximal in the presence of DBP at pH 8.5, 4% SOM, and 60% maximum WHC (3.0 g) and minima with DEHP at pH 8.5, 2% SOM, and 80% maximum WHC (0.2 g), indicating that the toxicity of DBP at a higher pH, higher SOM content, and 60% maximum WHC was significantly higher than that of DEHP at a higher pH, lower SOM content, and 80% maximum WHC (*p* < 0.05). In general, at pH 7.0, there was less inhibition of plant biomass in soil with DBP treatment, indicating that DEHP was more toxic. By contrast, at pH 8.5, there was much less inhibition of biomass in soil by DEHP, indicating that DBP was more toxic. The difference in phytotoxicity between DBP and DEHP was relatively low in soil at pH 7.0 compared with soils of higher pH values, suggesting that soil pH may be an important factor affecting the phytotoxicity of both PAE compounds.

### 3.2. Effects on Phytochromes

The plant pigment contents in water spinach treated by the two tested PAEs after 30 days of incubation are shown in Figure 1a. The declines in chlorophyll a content and carotenoid content showed similar trends, especially in treatments with 20 mg DBP kg^−1^, as shown by the significant differences compared to the controls (*p* < 0.05) and highly significant differences (*p* < 0.01) in Figure 1a. The results indicate higher phytotoxicity of DBP under different soil conditions. In the case of DBP, the decline in plant pigment concentrations was much higher under a higher SOM content and at 60% maximum WHC, indicating a higher toxicity of DBP in neutral soil with a higher SOM content and moderate soil water content compared with the other conditions. The decline in chlorophyll a content (Figure 1a) in water spinach treated with DBP was not always large but was often significant, while the decline in chlorophyll a content in DEHP treatments changed little, ranging from 1.8 to 2.3 mg g^−1^. By contrast, the decline in carotenoid content (Figure 1a) at higher SOM contents and 60% maximum WHC was steady and was significantly different (*p* < 0.05) from the controls. The decline in carotenoid content was three times as large with DBP at pH 7.0, 4% SOM, and 60% maximum WHC than with DEHP at pH 7.0, 2% SOM, and 80% maximum WHC.

### 3.3. Effects on Plant FAA Contents

The shoots were used for FAA analysis because they are the most commonly consumed plant part. The increases in FAA content in shoots under adjusted different soil conditions (shown in Figure 1b) indicate that the shoot FAA contents all increased, especially with DBP at pH 7.0, 2% SOM, and 80% maximum WHC (*p* < 0.05), which was the only treatment showing a significant difference from the control. All other treatments showed no significant increase in foliar FAA content over the controls. The lowest increases in FAA occurred with both DBP and DEHP at pH 8.5, 2% SOM, and 80% maximum WHC.

### 3.4. Effects on Plant Superoxide Anion Free Radical and Hydroxyl Radical Activity

The increase in superoxide anion free radical activity in the shoots of water spinach is shown in Figure 1b. The activity of the superoxide anion free radical was generally enhanced in all treatments, especially in the presence of DBP (*p* < 0.05), in a similar fashion to the FAA results. Only in alkaline conditions with lower SOM and higher water content was the shoot superoxide anion free radical activity significantly higher than in the control (*p* < 0.05). In all of the other DEHP treatments, there were no significant increases in superoxide anion free radical activity (*p* > 0.05). The increase in superoxide anion free radical activity under different soil conditions in the presence of DBP was about 1.4 times from the highest to lowest concentration, and about 3.6 times with the DEHP treatments. The maximum value (37.3 U L^−1^) was almost five times the minimum value (7.6 U L^−1^).

Increases in the shoot hydroxyl radical activity are displayed in Figure 1b. The hydroxyl radical activity was generally elevated in all treatments, especially at the lower SOM and higher water contents (*p* < 0.05). The maximum value (35.8 U mL^−1^) was almost 2.2 times the minimum (16.2 U mL^−1^).

### 3.5. Effects on the Soil Microbial Community

**Figure 2:**
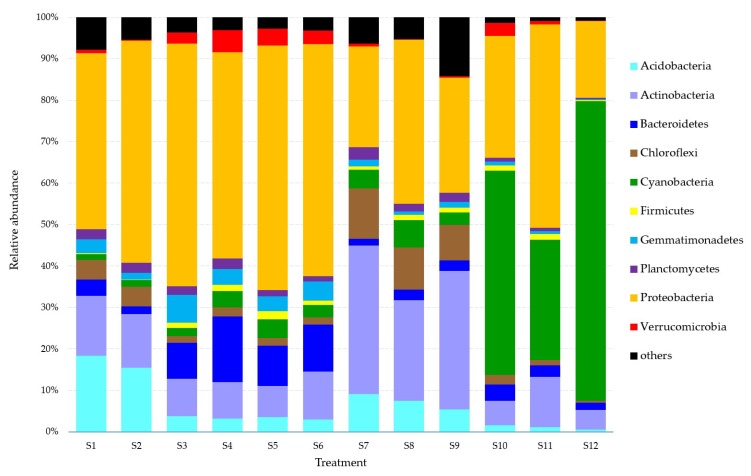
Relative abundance of bacteria at phylum level in different treatments (abundance > 1%). Refer to Figure 1 for treatment details. Each value is the mean of four replicates ± SEM.

Figure 2 shows the relative abundance of bacteria at the phylum level in the different treatments. Generally, compared within each group from the bar graph, DEHP at a concentration of 20 mg kg^−1^ at pH 7.0, 2.0% SOM, and 80% maximum WHC changed the soil microbial community to a greater extent than DBP, indicating that DEHP was more toxic under these soil conditions. DBP and DEHP at a concentration of 20 mg kg^−1^ at pH 8.5, 2.0% SOM, and 80% maximum WHC were more similar to each other and with DEHP at a concentration of 20 mg kg^−1^ at pH 7.0, 2.0% SOM, and 80% maximum WHC, suggesting that target PAE compounds have similar toxicity under conditions of 2.0% SOM and 80% maximum WHC at different pH values. However, for other treatments (S7–S12), no obvious regulation could be concluded based on the data of Figure 2, indicating the important impact of higher SOM on the toxicity of DBP and DEHP.

According to the determined data, the sequences of all groups could be classified into 19 phyla. Nine phyla with an abundance of ≤1% were excluded. Unclassified sequences that could not be classified into any known phylum were all grouped into “others”. The 10 dominant phyla were Acidobacteria, Actinobacteria, Bacteroidetes, Chloroflexi, Cyanobacteria, Firmicutes, Gemmatimonadetes, Planctomycetes, Proteobacteria, and Verrucomicrobia. Proteobacteria (ranging from 18.56% to 59.03%) comprised the predominant phylum in all treatments. From S1 to S12, other predominant phyla were Acidobacteria (control at pH 7.0, 2% SOM, 80% maximum WHC 22.44%; DBP at pH 7.0, 2% SOM, 80% maximum WHC 18.36%), Actinobacteria (DEHP at pH 7.0, 2% SOM, 80% maximum WHC 9.4%), Bacteroidetes (control at pH 8.5, 2% SOM, 80% maximum WHC 16.30% and DBP at pH 8.5, 2% SOM, 80% maximum WHC 10.03%), Actinobacteria (DEHP at pH 8.5, 2% SOM, 80% maximum WHC 11.92%; control at pH 7.0, 4% SOM, 60% maximum WHC 39.49%; DBP at pH 7.0, 4% SOM, 60% maximum WHC 26.26%; and DEHP at pH 7.0, 4% SOM, 60% maximum WHC 35.34%), and Cyanobacteria (control 50.10%, DBP 29.39%, and DEHP 72.70%, all at pH 8.5, 4% SOM, 60% maximum WHC) (Figure 2).

**Figure 3:**
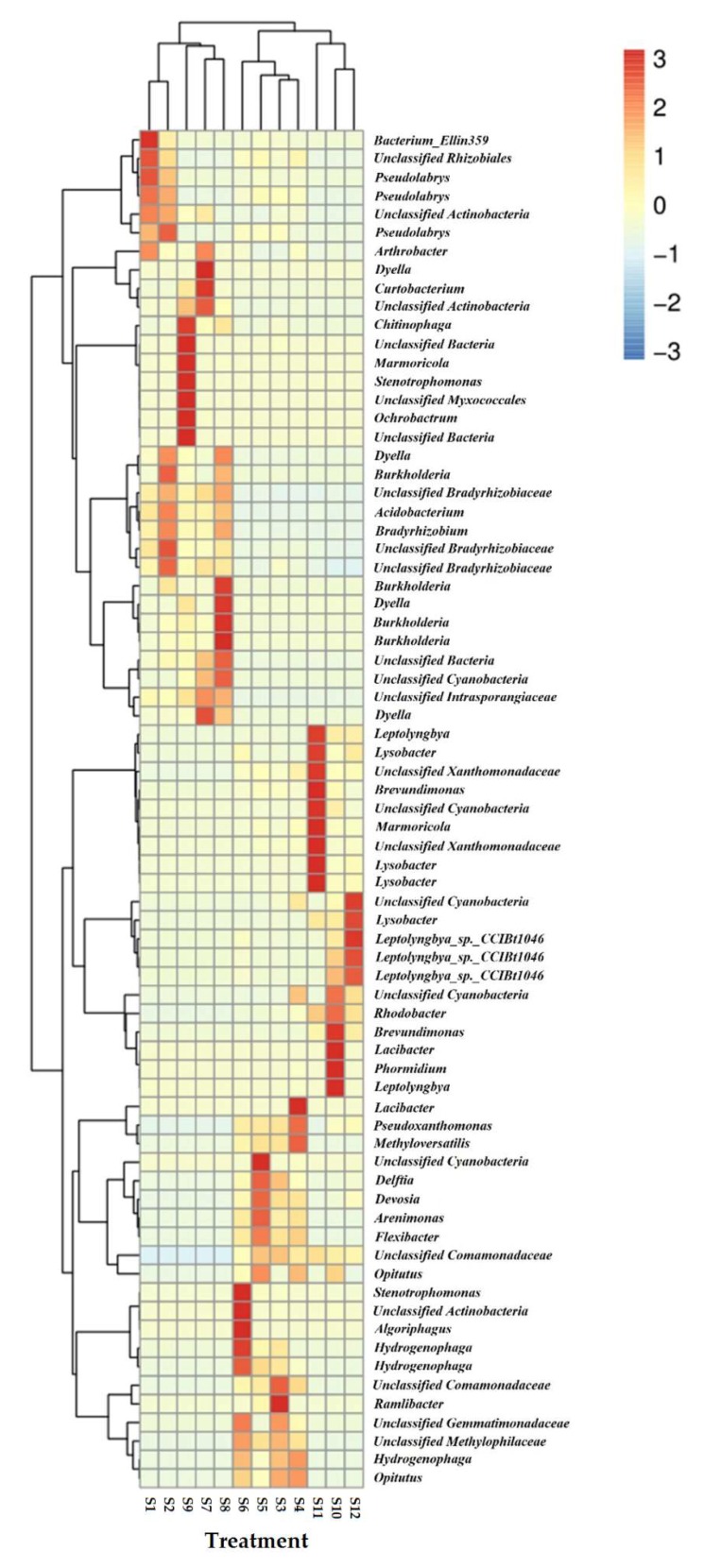
Heatmap of the relative abundance of the top 10 predominant genera in each treatment. Refer to Figure 1 for treatment details. Each value is the mean of four replicates ± SEM.

A heatmap of the 10 predominant genera in each treatment is exhibited in Figure 3. In each treatment, the dominant genus showed significant differences. *Dyella*, *Chitinophaga*, *Marmoricola*, *Stenotrophomonas*, *Ochrobactrum*, *Burkholderia*, *Leptolyngbya*, *Lysobacter*, *Brevundimonas*, *Lacibacter*, *Phormidium*, *Algoriphagus*, *Ramlibacter*, bacterium_Ellin359, and *Pseudolabrys* were found to be especially enriched under different treatments.

**Figure 4:**
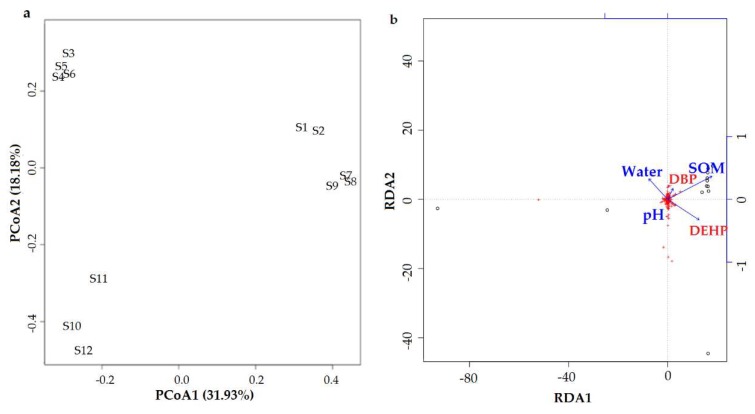
(**a**) PCoA (principal coordinate analysis) of bacterial OTUs (Operational Taxonomic Units) at the 97% level based on the Bray–Curtis distance in different treatments and (**b**) redundancy analysis (RDA) of different treatments in terms of three environmental factors. Refer to Figure 1 for treatment details. Each value is the mean of four replicates ± SEM. Notes: SOM: soil organic matter; DBP: di-*n*-butyl phthalate; DEHP: bis(2-ethylhexyl) phthalate.

The PCoA was based on the Bray–Curtis distance of OTUs at 97% cutoff, which was used to visualize the differences in the compositions of communities among different treatments (Figure 4a). PCoA implies that the bacterial composition of the 12 treatments was alike in soils with the same conditions and evidently differed under different soil conditions, except in treatments S1, S2, and S3. The contribution rate of the first principal component was 31.93%, which explained 31.93% of the alterations in the bacterial community composition. The contribution rate of the second principal component explained 18.18% of the alterations in the bacterial community composition. Treatments at the same soil condition with or without PAEs were compared to determine the effects of target PAEs. Treatments in two groups—(1) S4, S5, and S6; and (2) S7, S8, and S9—were closer to each other, indicating that the effects of DBP and DEHP under pH 8.5, 2.0% SOM, and 80% maximum WHC and pH 7.0, 4.0% SOM, and 60% maximum WHC were not as obvious as those under other soil conditions. Treatments with the same DBP or DEHP concentrations under different soil conditions (S1: 4, 7, 10; S2: 5, 8, 11; S3: 6, 9, 12) were compared to determine the effects of soil conditions. Only S3 and S6 were similar to each other, indicating that pH values are not the most significant factor related to the toxicity of DEHP to soil microorganisms.

The redundancy analysis (RDA) of different treatments regarding three environmental factors is shown in Figure 4b. The results indicate that the toxicity of DBP and DEHP was positively correlated with the SOM content and negatively correlated with the soil water content, with no clear correlation with soil pH values. DEHP and DBP were positively correlated with RDA1, DBP was positively correlated with RDA2, but DEHP was negatively correlated with RDA2.

The determined concentrations of target PAEs and soil parameters on days 0 and 30 are shown in Table 1. The calculated Chao, Ace, Shannon, and Simpson indices for 97% OTU clusters of control treatments in four groups were all significantly different from the other treatments (Table 2).

## 4. Discussion

### 4.1. Plant Biomass

Soil pH, oxygen availability, and nutrient status can affect the degradation of toxic pollutants, which is closely related to the toxicity of the soil target pollutants [44]. Plant biomass declined in the presence of DBP and most of the declines were considered significant (*p* < 0.05) when compared with the controls, suggesting that biomass may be a suitable indicator of the toxicity of DBP to water spinach. Under neutral soil conditions, DBP exhibited lower toxicity than DEHP, but under alkaline soil conditions, higher toxicity of DBP was observed. Thus, soil pH value has a large effect on DBP and DEHP toxicity to water spinach. However, compared with our former study on lettuce, in most treatments, plant biomass was promoted by the two target PAE compounds, especially under alkaline conditions (*p* < 0.01) [29]. For water spinach, the decrease of biomass under different pH conditions was more highly correlated with the type of target PAE compound. The disparity indicates the different mechanisms between plants when facing environmental stress, and the necessity of selecting multiple test organisms in ecotoxicity tests.

It has been reported that the efficiency of the abiotic degradation of PAEs with relatively short alkyl chains, such as DBP, is much lower at a neutral pH than in acid or in alkaline soil conditions. Neither hydrolysis nor photolysis of DEHP proceeds significantly at any pH, and hydrolysis at neutral pH is negligible [45]. However, the similarity between DEHP at pH 8.5 and both SOM contents and WHC contents indicates that the higher water content may have surrounded the DEHP with soil particles and thereby restricted its toxicity. As Table 1 shows, the degradation rates of both target pollutants were low (<30%), even under different pH conditions.

The degradation and adsorption behavior of the PAE compounds differed in neutral or alkaline soils. For example, with its higher molecular weight and structural complexity, DEHP could usually avoid biotransformation in comparison with the lower molecular weight DBP. Moreover, the bioavailability and toxic effects of target PAEs are influenced by their lipotropy, which is related to SOM content [46]. The lower biomass inhibition of the test plant in DEHP treatments under alkaline soil conditions might be ascribed to its higher adsorption and lower bioavailability. The higher toxicity of DBP may have been observed because the toxic effects of DEHP are vulnerable to external soil conditions. The water content and organic matter content are also critical factors because they can affect the toxicity of target pollutants by changing the balance between adsorption and desorption.

### 4.2. Phytochromes

Chlorophyll a and b and carotenoids are important metabolites and components of photosynthesis. Decreases in the content of chlorophyll a and carotenoids showed similar trends, especially in treatments with 20 mg DBP kg^−1^, revealing significant differences with the control treatments (*p* < 0.05) and highly significant differences (*p* < 0.01) in Figure 1a. The results indicate a higher toxicity of DBP to water spinach under the experimental soil conditions. The chlorophyll a content decline (Figure 1a) in water spinach treated with DBP was not always large or statistically significantly different, and the results suggest the importance of soil pH in affecting the toxicity of DBP. The decline in chlorophyll a and carotenoid contents (Figure 1a) in the presence of DEHP also highlights the importance of soil pH in influencing the phytotoxicity of DEHP. The results of chlorophyll a and carotenoids agree with that of lettuce, even at different SOM and soil water contents [29]. The comparison of results between lettuce and water spinach also indicates that lettuce is more sensitive to the toxicity of single DEHP, because extremely significant differences were observed for phytochrome indices (*p* < 0.01) at the spiked concentration of 20 mg kg^−1^ [29]. However, in water spinach, differences compared with corresponding controls were more obvious for DBP (*p* < 0.01), indicating that water spinach is more sensitive and suitable as an indicator of the presence of DBP.

Changes in chlorophyll content take place at the molecular level in cells and occur much earlier than any observed growth inhibition [47], so chlorophyll content has been recommended as a biomarker in plant cellular systems exposed to xenobiotics [48]. However, when vegetables such as lettuce are used for PAE remediation, the biomass and chlorophyll of the plant leaves would normally be increased [49]. Carotenoids are non-enzymatic antioxidants that protect chlorophyll molecules from oxidative stress and delay the aging process of healthy cells [50], and can protect human cells from mutagens in consumed vegetables. Carotenoids are often analyzed as their profiles can provide substantial evidence in rationalizing the response of plants in terms of their growth characteristics and symptoms [51]. Chlorophyll a and b and carotenoid contents in germinating onions were inhibited by both single DBP and single DEHP [16]. In the current experiment, the carotenoid content in test water spinach leaves showed a decreasing trend, indicating reduced defense against free radicals and declines in nutritional value and quality.

### 4.3. FAA Content, Superoxide Anion Free Radical Activity, and OH^−^ Activity

FAA content is a very sensitive factor reflecting the toxicity of PAEs. For example, whole plants of mung bean FAA under DBP treatment tended to be stimulated significantly (*p* < 0.01) [52]. However, in water spinach seedlings, the only significant change observed (*p* < 0.05) was in the presence of DBP at pH 7.0, 2% SOM, and 80% maximum WHC. The consumption of protein and a decline in protein synthesis could be seen as the resistance of the plant under different environmental pressures [16]. Conclusions in early reports pointed out that the FAA contents in tissues will be promoted when plants are subject to drought, high salinity, improper temperatures, and other adverse environmental conditions. Even low concentrations of toxic substances such as trifluralin could promote the level of FAAs in muskmelon seedlings [53]. In the present study, the accumulation of FAAs also reflects the adverse impacts of the target pollutants and especially DBP.

Differences in toxicity between DBP and DEHP can be seen in terms of the significant differences in the increasing superoxide anion free radical activity in the presence of DBP at pH 7.0 and 8.0, 2% and 4% SOM, and 80% and 60% maximum WHC (*p* < 0.05 or *p* < 0.01), indicating a higher toxicity of DBP to water spinach. This is also supported by the results of the DEHP treatments, as shoot superoxide anion free radical activity was significantly increased by DEHP alone at pH 8.5, 2% SOM, and 80% maximum WHC (*p* < 0.05). Shoot superoxide anion free radical activity was slightly sensitive to soil pH in both DBP and DEHP treatments. Incomplete reduction of oxygen in the process of aerobic metabolism during the growth and development of higher plants will lead to greater production of reactive oxygen species (ROSs), including the superoxide anion free radical, hydroxy radical, singlet oxygen, and hydrogen peroxide. The existence of ROSs is due to the failure of the reduction of oxygen to form water, which can result in strong oxidation damage to plants. In the present study, hydroxyl radical activity appeared to be generally promoted in all treatments, but only significantly under alkaline conditions with both PAEs at 2% SOM and 80% maximum WHC (*p* < 0.05). The excited oxygen molecule decomposes into ROSs, excited states of bimolecular oxygen, and the superoxide anion radical. The latter decomposes to H_2_O_2_ and then to hydroxyl radicals. All ROSs react with drug molecules, leading to their degradation [54]. Total superoxide dismutase (T-SOD) can convert the superoxide radical anion to hydrogen peroxide and water while catalyzing its dismutation to act as the first-line defense barrier against oxidative stress [55]. The superoxide anion radical may lead to severe membrane structural damage and damage to photosynthetic pigments and the photosynthetic system [56], indicating the reaction of the plant to environmental stress. Under adverse environmental conditions, the ability to clear active oxygen may be abolished when the concentration of active oxygen free radicals becomes excessive [57]. In the current experiment, there was a clear promotion (*p* < 0.05) of superoxide anion free radical activity in DBP treatments, especially under alkaline soil conditions, indicating damage from higher toxicity and the production of oxygen free radicals. In lettuce, superoxide anion free radical activity was also shown to be promoted more in alkaline soils (*p* < 0.01) [29]. The superoxide anion free radical activity of water spinach is more sensitive to DBP (*p* < 0.05) and could be used as a bioindicator for DBP contamination.

### 4.4. Effects on the Soil Microbial Community

Figure 2 shows that the 10 dominant phyla detected across the 12 treatments were Acidobacteria, Actinobacteria, Bacteroidetes, Chloroflexi, Cyanobacteria, Firmicutes, Gemmatimonadetes, Planctomycetes, Proteobacteria, and Verrucomicrobia. Proteobacteria, the predominant phylum in all 12 treatments (ranging from 18.56% to 59.03%), is usually isolated from contaminated soils as it is capable of degrading aromatic compounds and may be used as a potential biological indicator of multiple contaminations by polycyclic aromatic hydrocarbons in agricultural soils [58]. Actinobacteria (the second most common phylum detected), Acidobacteria, Bacteroidetes, and Cyanobacteria are commonly found in soils. Actinobacteria are associated with the remediation of trace metal contamination [59]. Studies show that filamentous Gram-positive Actinobacteria are involved in the bioremediation of the potentially toxic metals copper, chromium, cadmium [60], lead [61], and zinc [62]. This is because many genera of actinobacteria are able to survive under extreme conditions, such as high temperatures, low moisture, and nutrient starvation, to produce biosurfactants that facilitate biodegradation processes [63]. Actinobacteria have also been isolated from pesticide-contaminated areas due to their tolerance of lindane and their ability to use pesticides as a sole carbon source [64]. Compared with the well-understood phylum Proteobacteria, the phylum Acidobacteria is a metabolically and genetically diverse group [65,66]. However, due to their advantages in number and metabolic activity, bacteria belonging to the Acidobacteria phylum might be crucial for the biogeochemical cycles of rhizosphere soils [67]. Acidobacteria are known to dominate less contaminated environments, while Bacteroidetes are more abundant in environments with heavy pollution, regardless of substratum types (soil or excavated gravel material) [68]. Changes in soil microbial community structure due to petroleum pollution in northern Shaanxi province have been investigated using high-throughput sequencing to analyze the soil microbial community structures in soil samples. The results indicate that the Bacteroidetes and Proteobacteria community contents increased in representation while the Acidobacteria and Gemmatimonadetes community contents decreased with increasing petroleum hydrocarbon content [69]. Apart from being abundant members of the soil microbiota, members of the phylum Bacteroidetes are also well known to be dominant intestinal microbiota [70]. However, there is little information about the potential persistence of fecal Bacteroidetes populations in soils or other extraintestinal environments [71]. One of the most negative consequences of eutrophication is cyanobacteria proliferation and toxin release to surface waters [72]. However, microscopic algae and cyanobacteria are permanent components of terrestrial phytocenoses, where they form phototropic blocks of soil microbial cenoses and are dwellers in either natural or anthropogenically disturbed soils [73].

The taxonomic analysis at the genus level using the heatmap showed that the dominant bacteria differed significantly between treatments (Figure 3). In addition to the 18 classified enriched bacteria, unclassified cyanobacteria were found in the soils of most of the treatments at pH 8.5. Cyanobacteria have been suggested for use in reclaiming calcareous soils because they form a thick stratum on topsoil during the rainy season and in the winter [74]. Cyanobacterial growth has been found to markedly decrease the pH of calcareous soils under natural conditions [74], and this may be one explanation for the high buffering capacity of soils against extremes of acidity or alkalinity. In the present study, the soil pH was increased by adding calcium hydroxide. The detection of unclassified cyanobacteria might provide a hint of a cumulative effect in the reclamation of calcareous soils. Special functions of the 18 enriched bacteria have been detected in previous studies. *Dyella ginsengisoli* LA-4 has been found to degrade a range of aromatic compounds, including biphenyls [75]. However, prior to being isolated from activated sludge, no reports had been published on the genus *Dyella* regarding environmental pollution biodegradation. The occurrence of this degradation ability in the genus *Dyella* has led to its use as a new microbial resource in environmental bioremediation [76]. *Dyella* was enriched in three of the treatments, especially with DBP at pH 7.0, 4% SOM, and 60% maximum WHC. *Burkholderia* was also greatly enriched in the presence of DBP at pH 7.0. Many *Burkholderia* species could be used in biological control, bioremediation, atmospheric nitrogen fixation, plant growth stimulation, and other applications. Moreover, their potential uses could be even greater because of their ability to colonize the rhizospheres of maize, wheat, rice, grasses, oat, lupine, coffee, and other plants at high population densities [77]. The occurrence of *Burkholderia* in DBP treatments at pH 7.0 indicates that SOM content and soil moisture level are not constraints to this genus.

The results of 454 high-throughput pyrosequencing indicated the differentiation of the bacterial community structure between set treatments in the PCoA plot based on the OTU composition at the 97% level (Figure 4a). However, the bacterial community structure differentiation between groups was more significant than among treatments. The RDA results (Figure 4b) indicate that the toxicity of DBP and DEHP was positively correlated with the SOM content, but negatively correlated with the soil water content, with no clear relationship with soil pH, in contrast to the plant results. From the results of the Mental test in Table S2, the beta diversity of bacterial communities is more significant correlated with SOM. The rhizobacteria form a group of the most adapted microorganisms [78,79]. Rhizobacteria often play important roles in increasing crop productivity, are known to display many functions ranging from plant growth promotion to soil nutrient recycling, and have been investigated as possible replacements for chemical fertilizers in soils, showing a great diversity in chemical, physical, and biological properties [80].

The bacterial richness estimators (Chao and Ace) and diversity indices (Shannon and Simpson) calculated for the 97% OTU clusters for four control treatments were significantly different (Table 2), indicating a marked change after the addition of DBP or DEHP to the soil. The differences in each group compared with the controls indicate the importance of the toxicity of the two PAE pollutants, irrespective of the soil conditions. In our former study on lettuce, the Shannon and Simpson indices decreased when the target PAEs were added, and the addition of DBP led to higher inhibition of microbial diversity [29]. However, in the present study, only the Shannon indices decreased as PAE compounds were added. The Simpson indices showed extremely significant differences (*p* < 0.01) in treatments with PAEs compared with the corresponding control treatments, which made them more suitable for use as an indicator, due to their sensitivity. However, the Simpson index was only inhibited in S11 (DBP 20 mg kg^−1^ at pH 8.5, 4.0% SOM, 60% maximum WHC), which also demonstrated the higher toxicity of DBP, similar to our previous study.

## 5. Conclusions

Soil pH is likely to be a major factor affecting plant physiology, while the soil microbial community is more sensitive to SOM content. DBP was shown to be more toxic at a lower SOM content and higher soil moisture level under alkaline conditions, and at a higher SOM content and lower soil moisture level in neutral soil. The toxicity of DEHP was higher at a lower SOM content and higher soil moisture level in neutral soil, and at a higher SOM content and lower soil moisture level in alkaline soil. Compared with lettuce, water spinach is more sensitive to the toxicity of DBP than DEHP. Examining the combined responses of the plants and microorganisms gives a more accurate overview when assessing the toxicity effects of PAE pollutants under different soil conditions. It is important to clarify the main soil factors affecting the toxic effects of the most common typical PAE compounds under different soil conditions in Chinese greenhouse soils because of their soil diversity and complexity. The results can be used to reduce the toxic effects of target pollutants, to design management systems for the safe production of vegetables for human consumption, to direct the agricultural use of plastic mulching films, and for the remediation of soils polluted with PAEs.

## Figures and Tables

**Table 1:** Controlled variable results from the experimental treatments at the start and end of the toxicity test.

Treatment		Controlled Variables				Day 0				Day 30			
		PAE Con. (mg kg^−1^)	SOM Content (%)	Water Content (% Max WHC)	pH	PAE Conc. (mg kg^−1^)	SOM Content (%)	Water Content (% Max WHC)	pH	PAEs Conc. (mg kg^−1^)	SOM Content (%)	Water Content (% Max WHC)	pH
Group 1	S1	0 Control	2.0	80	7.0	-	1.9 ± 0.3	82 ± 2	6.5 ± 0.4	-	1.6 ± 0.2	85 ± 3	6.7 ± 0.3
	S2	20 DBP				19.8 ± 0.4	1.8 ± 0.3	77 ± 5	7.0 ± 0.2	15.6 ± 0.5	1.5 ± 0.3	83 ± 5	7.2 ± 0.3
	S3	20 DEHP				20.3 ± 0.2	1.9 ± 0.3	79 ± 4	6.9 ± 0.2	17.2 ± 0.4	1.7 ± 0.2	85 ± 4	7.1 ± 0.2
Group 2	S4	0 Control			8.5	-	2.0 ± 0.2	76 ± 3	8.2 ± 0.2	-	1.7 ± 0.2	86 ± 2	8.3 ± 0.1
	S5	20 DBP				19.1 ± 0.5	2.0 ± 0.4	83 ± 4	8.5 ± 0.3	14.3 ± 0.4	1.8 ± 0.4	86 ± 3	8.4 ± 0.2
	S6	20 DEHP				19.7 ± 0.4	2.0 ± 0.3	79 ± 5	8.2 ± 0.2	15.5 ± 0.5	1.7 ± 0.3	83 ± 3	8.4 ± 0.3
Group 3	S7	0 Control	4.0	60	7.0	-	3.6 ± 0.2	63 ± 5	7.1 ± 0.3	-	3.3 ± 0.4	58 ± 3	7.3 ± 0.4
	S8	20 DBP				18.7 ± 0.5	3.9 ± 0.4	58 ± 3	7.1 ± 0.2	15.9 ± 0.3	3.6 ± 0.2	56 ± 5	7.2 ± 0.3
	S9	20 DEHP				19.9± 0.3	4.2 ± 0.3	56 ± 4	6.8 ± 0.3	16.8 ± 0.3	4.0 ± 0.3	55 ± 3	6.9 ± 0.3
Group 4	S10	0 Control			8.5	-	4.4 ± 0.2	59 ± 2	8.0 ± 0.2	-	4.1 ± 0.3	56 ± 2	8.2 ± 0.3
	S11	20 DBP				19.3 ± 0.6	3.9 ± 0.3	62 ± 4	8.1 ± 0.2	14.6 ± 0.3	3.5 ± 0.2	55 ± 4	8.4 ± 0.4
	S12	20 DEHP				20.5 ± 0.3	3.7 ± 0.4	60 ± 2	8.8 ± 0.4	16.8 ± 0.5	3.4 ± 0.2	56 ± 3	8.9 ± 0.3

Values are the average of four replicate pots ± the standard error of the mean (SEM) in each treatment. “-”, total phthalate acid ester (PAE) concentration <0.15 mg kg^−1^. DBP: di-*n*-butyl phthalate; DEHP: bis(2-ethylhexyl) phthalate; SOM: soil organic matter; WHC: water-holding capacity.

**Table 2:** Diversity indices and richness of bacteria in the different treatments.

Treatment		Ace	Chao	Shannon	Simpson	Coverage
Group 1	S1	5596	4344	7.10	0.0016	0.80
	S2	4854 **	3664 **	6.84 **	0.0022 **	0.83
	S3	4585 **	3422 **	6.64 **	0.0033 **	0.87
Group 2	S4	4258	3263	6.75	0.0028	0.84
	S5	4042 *	3019 *	6.53	0.0033 **	0.86
	S6	4183	3202	6.41	0.0052 **	0.88
Group 3	S7	5331	4163	7.06	0.0026	0.83
	S8	4218 **	3368 **	6.69 *	0.0035 **	0.88
	S9	4571 **	3588 **	6.36 **	0.0106 **	0.89
Group 4	S10	2517	1982	4.77	0.0934	0.92
	S11	2228 **	1722 **	4.62	0.0398 **	0.95
	S12	1985 **	1505 **	3.03 **	0.3249 **	0.96

Refer to Figure 1 for treatment details. * significantly different at *p* < 0.05; ** significantly different at *p* < 0.01 according to a Duncan’s multiple range test within each group compared with the controls

## Supplementary Materials

The following are available online at https://www.mdpi.com/1660-4601/16/18/3489/s1: Table S1. Recovery results of the CRMs in method quality control; Table S2. Correlations between detected OTUs and environmental parameters assessed by Mantel test.
