# Supplementary material for: Effects of Phthalate Esters on Ipomoea aquatica Forsk. Seedlings and the Soil Microbial Community Structure under Different Soil Conditions
Source: Int J Environ Res Public Health. 2019 Sep 19;16(18):3489. doi: 10.3390/ijerph16183489 (PMC6766064; doi:10.3390/ijerph16183489)
Supplement: Supplementary file 1 [file ijerph-16-03489-s001.pdf]

Supplementary Material

Table 1. Recovery results of the CRMs in method quality control.

**Compound**	**Given Confidence Interval (mg kg^{-1})**		**Determined Values (mg kg^{-1})**	
	**CRM 136–100**	**CRM 119–100**	**CRM 136–100**	**CRM 119–100**
DBP	0.64 − 0.80	Not exist	0.73 ± 0.08	Not detected
DEHP	0.82 − 0.96	7.65 − 9.94	0.92 ± 0.11	9.38 ± 0.17

Table 2. Correlations between detected OTUs and environmental parameters assessed by Mantel test.

**Parameter**	**Mantel Statistic *r***	**Significance**
DBP	−0.122	0.893
DEHP	−0.03643	0.528
SOM	0.08358	0.042
Water content	0.1426	0.136
pH	0.1426	0.133
